# Treadmill exercise suppressed stress-induced dendritic spine elimination in mouse barrel cortex and improved working memory via BDNF/TrkB pathway

**DOI:** 10.1038/tp.2017.41

**Published:** 2017-03-21

**Authors:** K Chen, L Zhang, M Tan, C S W Lai, A Li, C Ren, K-F So

**Affiliations:** 1 Guangdong-Hong Kong-Macau Institute of CNS Regeneration, Jinan University, Guangzhou, China; 2 Guangdong Medical Key Laboratory of Brain Function and Diseases, Jinan University, Guangzhou, China; 3 Guangdong-Hong Kong-Macau Collaboration and Innovation Center for Tissue Regeneration and Repair, Jinan University, Guangzhou, China; 4 Co-Innovation Center of Neuroregeneration, Nantong University, Jiangsu, China; 5 School of Biomedical Sciences, The University of Hong Kong, Hong Kong SAR, China; 6 Department of Ophthalmology and State Key Laboratory of Brain and Cognitive Sciences, The University of Hong Kong, Hong Kong SAR, China

## Abstract

Stress-related memory deficit is correlated with dendritic spine loss. Physical exercise improves memory function and promotes spinogenesis. However, no studies have been performed to directly observe exercise-related effects on spine dynamics, in association with memory function. This study utilized transcranial two-photon *in vivo* microscopy to investigate dendritic spine formation and elimination in barrel cortex of mice under physical constrain or naive conditions, followed by memory performance in a whisker-dependent novel texture discrimination task. We found that stressed mice had elevated spine elimination rate in mouse barrel cortex plus deficits in memory retrieval, both of which can be rescued by chronic exercise on treadmill. Exercise also elevated brain-derived neurotrophic factor (BDNF) expression in barrel cortex. The above-mentioned rescuing effects for both spinognesis and memory function were abolished after inhibiting BDNF/tyrosine kinase B (TrkB) pathway. In summary, this study demonstrated the improvement of stress-associated memory function by exercise via facilitating spine retention in a BDNF/TrkB-dependent manner.

## Introduction

Stress is one major concern affecting mental health of general population, and leads to a series of cognitive disorders including memory deficits.^1^ Scientists have paid long-term efforts to elucidating the mechanism underlying stress-induced memory impairment. Recently dendritic spine dynamics including formation, elimination and retention have been studied regarding its correlation with memory function. Stress can induce dendritic spine loss in prefrontal cortex,^2^ amygdala^3^ and hippocampus.^4^ Such effects are likely to be caused by neurotrophic factors such as brain-derived nerve growth factor (BDNF).^5^ The suppressed spinogenesis in hippocampus may further contribute to stress-induced memory deficit.^6^ Our previous findings showed the improvement of hippocampal neurogenesis and depression-like behavior by physical exercise.^7^ However, it is still unclear whether exercise has similar antidepressant effect in cortical regions.

The role of physical exercise on memory retention and learning has been recognized on both animal models^8^ and humans.^9, 10^ Abundant researches have revealed beneficial effects of exercise on memory function possibly via facilitating local neurogenesis and long-term potentiation in hippocampus,^11, 12^ in addition to functional recovery in patients of Parkinson's^13^ or Alzheimer's.^14^ The cellular and molecular mechanism underlying exercise-induced memory improvement is still inconclusive at the current stage. Spine plasticity provides one possible explanation as stably maintained dendritic spines are necessary for memory function.^15^ Physical exercise has been shown to increase spine density and synaptogenesis in hippocampal, cortical and cerebellar neurons,^16, 17, 18^ and prevented spine loss in Parkinson's mouse.^19^ The study of spine plasticity thus may provide more evidences for mechanism of exercise-induced memory improvement. Spine dynamics of sensory cortex is tightly correlated with sensory inputs in a region-specific manner.^20, 21, 22, 23^ Barrel cortex is one region of somatosensory cortex responsible for whisker-dependent learning and memory functions in rodents. The spine plasticity of barrel cortex is tightly correlated with whisker-related sensory inputs, as the deprivation of sensory inputs by whisker trimming disrupted spine plasticity and receptive field structure,^21, 24^ whereas whisker-dependent learning stabilized preexisting spines.^25, 26^ Barrel cortex thus provides a good model for studying spine plasticity and memory function. No direct study, however, has been performed to observe the effect of physical exercise on sensory regions including barrel cortex. The molecular mechanism connecting exercise and spinogenesis in neocortex is also lacking.

To determine the regulatory paradigm of physical exercise on memory function and spine dynamics in the cortical region, we used whisker-dependent memory task coupled with transcranial two-photon microscopy to track spine dynamics of apical tuft of layer V pyramidal neurons in the barrel cortex and memory retention after treadmill exercise on physical-induced-stress mice. We found decreased elimination and enhanced stability of dendritic spines in the barrel cortex with exercise, in addition to improved whisker-associated memory under either stress or normal conditions. Furthermore, the effects of exercise on modulating dendritic spine plasticity and behavior were found to be dependent on BDNF/tyrosine kinase B (TrkB) pathway.

## Materials and methods

### Experimental animals and grouping

The male Thy1-H line mice, which express yellow fluorescent protein in pyramidal neurons of cortical layer V were purchased from Jackson Laboratory (Bar Harbor, ME, USA) and were bred in-house. All the animals were group-housed under normal light–dark cycle (12 h/12 h) with food and water *ad libitum*. All experimental protocols have been pre-approved by the Laboratory Animal Ethics Committee at Jinan University in accordance with National Guidance for Animal Experiment.

In stress model study, the animals were divided into sedentary group, stress group and stress+exercise group, the latter two of which were prepared for stress model using physical restrain approach. In the study on naive mice, the animals were assigned into sedentary and exercise groups. In mechanism study, the animals were randomly divided into sedentary+DMSO (dimethyl sulfoxide) group, exercise+DMSO group and exercise+ANA-12 group. Animals with significant abnormalities in development, motility or mental status were excluded from all the experiments.

### Treadmill exercise

The treadmill exercise was performed according to previous literature.^27^ A treadmill apparatus for mice (Model JD-PT, Jide Instrument, Shanghai, China) was used for chronic physical exercise of mice. During the test session, the mice were allocated in the test room for 30 min acclimation, followed by 1 h continuous exercise at a velocity of 12 m min^−1^. Those mice that escaped from or refused the test were excluded from the experimental cohort. The exercise was carried out at 1400 h on each day.

### Physical stress model

A stress model was generated by physical restrain as previous described.^2^ The mouse was restrained for 1 h daily (1400–1500 h) in a plastic restrainer for 14 consecutive days. After restrain treatment, the mice were returned to their own cages. All the procedures were performed before physical exercise.

### Open-field test

The mouse was placed into a plastic box (50 cm × 50 cm) for freely exploring the arena during a single 10 min session. Movement paths were captured by an infrared digital camera. Ethovison XT version 3.0 (Noldus, Wageningen, The Netherlands) software was used to analyze the time duration of mouse in the arbitral defined center zone (25 cm × 25 cm) to evaluate its anxiety-like behavior.

### Elevated plus maze test

The apparatus for elevated plus maze consisted of two opposing open arms (44 cm length × 12 cm width) and two closed arms (44 cm length × 12 cm width), which were connected by a central zone (12 cm length × 12 cm width). The whole apparatus was elevated 50 cm above the floor. The mouse was placed in the center zone facing towards the open arm, and was allowed to freely explore the arena during a 5 min test session. Ethovision XT software was used to analyze the ratio of time duration in the open arm for evaluating anxiety-like behavior.

### Whisker-dependent novel discrimination task

To test the working memory related with whisker sensory inputs, the novel discrimination task was used as previously reported.^28^ The whole task consisted of two adaption sessions (on day 1 and day 2), and one learning phase followed by one test phase (both on day 3). The test arena was a plastic box (50 cm × 50 cm × 50 cm) filled with standard bedding (2 cm thickness) at the bottom. During the adaption session, the mouse was singly placed into the center of the test arena for 30 min acclimation. During the learning phase, two identical plastic plates (4 cm width, 15 cm high, 0.5 cm thickness) with commercially purchased sandpaper (80 grit) on both sides were placed in the center of the test arena. The mouse was allowed to freely explore the test apparatus for a fixed time period. After the learning phase, the mouse was given a rest in their home cage at different time intervals, followed by the test phase, in which one novel texture (identical size but with 220 grit sandpaper on both sides) was introduced to replace one of the original texture. Both textures were cleaned with 70% ethanol to remove any olfactory cues. The test room was illuminated by dim light and was kept quiet. A digital infrared camera (HDR-PJ675, SONY, Tokyo, Japan) was connected to the computer. Ethovison software was used to record movement path and data analysis. The approaching of objects was defined as entry within 4 cm around the texture.

### Real-time quantitative PCR

Total RNA was extracted from mouse barrel cortical tissues using Trizol reagent (Sangon Biotech, Shanghai, China) following the manual instruction. The complementary DNA was then synthesized by reverse transcription kit (Takara, Shiga, Japan). Real-time PCR was performed by SYBR Green reagent (Takara) using specific primers (BDNF-forward: 5′-TCATACTTCGGTTGCATGAAGG-3′, BDNF-reverse: 5′-AGACCTCTCGAACCTGCCC-3′ TrkB-forward: 5′-TGGTGCATTCCATTCACTGT-3′, TrkB-reverse: 5′-CGTGGTACTCCGTGTGATTG-3′ GAPDH-forward: 5′-AGGTCGGTGTGAACGGATTTG-3′, GAPDH-reverse: 5′-TGTAGACCATGTAGTTGAGGTCA-3′). RT-PCR was performed on a fluorescent quantitative PCR cycler (Model Eco-100-1004, Illumina, San Diego, CA, USA) under the following conditions: 95 °C denature, 30 s; followed by 40 cycles each containing 90 °C for 15 s, 60 °C for 30 s and 95 °C for 15 s. Transcriptional levels of *BDNF* and *TrkB* genes were normalized to housekeeping gene *GAPDH*. Quantitative analysis was performed by 2^−ΔΔ^^CT^ method.

### Western blotting

Total proteins were extracted from cortical tissues. BCA reagent (Beyotime, Shanghai, China) was used to measure the concentration of extracted proteins. A total of 10 μg protein sample was loaded for polyacrylamide gel electrophoresis (10% gel for TrkB, 12% gel for BDNF), and were transferred to polyvinylidene fluoride membrane (Pall Life Science, Port Washington, NY, USA) in a 200 V electrical field for 30 min. The membrane was first blocked for 1 h using 5% defatted milk powder, and then was incubated with rabbit anti-BDNF (1:2000, Abcam, Cambridge, MA, USA), goat anti-TrkB (1:1000, R&D System, Minneapolis, MN, USA) or rabbit anti-tubulin (1:2000, Abcam) primary antibody at 4°C overnight. Excess antibody was then washed by PBST, followed by incubation in horseradish peroxidase (horseradish peroxidase-conjugated goat anti-rabbit IgG (1:8000, ServiceBio, Wuhan, China) or mouse anti-goat IgG (1:10 000, ServiceBio) for 1 h at room temperature. The membrane was developed in ECL chromogenic substrates, and was imaged by an automatic protein imaging system (Bio-Rad, Hercules, CA, USA). Image J (NIH, Bethesda, MD, USA) software was used to measure optical density values of each band, which was normalized to that of tubulin. Each experiment was performed in triplicates.

### *In vivo* transcranial two-photon imaging

Dendritic spines were observed on living animals using the technique of thinned skull coupled with *in vivo* two-photon imaging.^29^ The mouse was anesthetized with 1.25% avertin (0.2 ml per 10 g, Sigma, St. Louis, MO, USA) and the head was fixed in a stereoscopic apparatus. The hair over the skull was shaved, and both eyes were sealed with eye ointment. After focal sterilization, a vertical incision was made along the midline, followed by gentle cleaning of connective tissues to expose the skull. With artificial cerebrospinal fluid washing, the barrel cortex was located (Bregma: −1.1 mm; midline: 3.4 mm) under the stereo-microscope (Stemi DV4, Zeiss, Oberkochen, Germany). A double-sided blade was then glued above the barrel cortical region. A high-speed surgical drill was used to create a thin-skull observing window with 0.5 mm diameter. The skull was further trimmed by a microsurgical blade.

The animal was then fixed on a customized stage for mouse under two-photon microscope (LSM780, Zeiss). A water-immersed objective ( × 20, 1.1 NA, Zeiss) was applied for observation. Yellow fluorescent protein fluorescence was excited by 920 nm laser. High-magnification images were scanned in a Z-stack manner (thickness: 80–120 μm; layer interval: 0.75 μm). The vascular structure of observing field was recorded using the built-in CCD camera, for the ease to localize identical area in next imaging. After imaging, the skull holder was gently detached and remaining glue was cleaned. Surgical silk was used to close the scalp. The mouse was singly housed during recovery and was returned to its home cage.

The captured images of dendritic spines were analyzed by Image J software. For quantifying formation and elimination rate, the spines were divided into two groups: filopodia with long protrusions but clearly head, and normal spines with visible heads. Furthermore, the spines were classified into four groups as previously described:^30^ mushroom, stubby, thin and filopodia. All analysis of spines was conducted by an independent observer in a blinded manner.

### Data analysis

All data were shown as mean±s.e.m. Statistical analysis was performed by GraphPad Prism software (GraphPad Software, La Jolla, CA, USA) by persons who were blinded to the animal grouping/treatment. Variance between groups were compared by F-test. Student's *t*-test was used to compare means between two groups, whereas one-way analysis of variance was used to compare means among multiple groups, followed by Tukey's *post hoc* test. A statistical significant level was defined when *P*<0.05.

## Results

### Stress-induced anxiety behavior and spine loss in barrel cortex can be rescued by physical exercise

**Figure 1:**
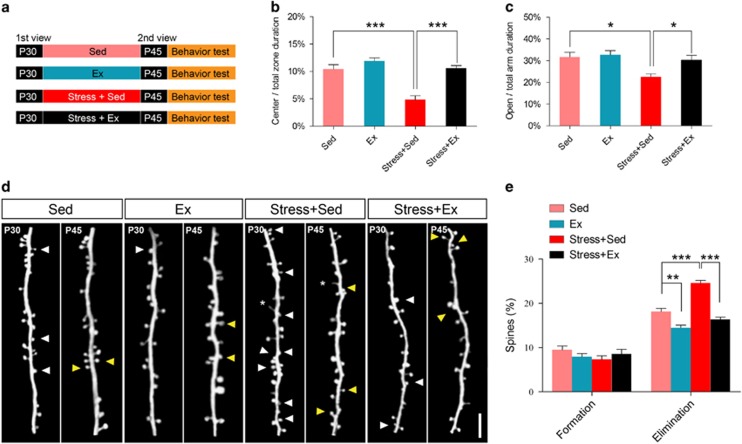
Physical exercise alleviated stress-induced anxiety behavior and dendritic spine loss. (**a**) Schematic diagram showing stress induction by physical constrain and treadmill exercise (Ex, 1 h per day, 12 m min^−1^) or sedentary housing (Sed) for 14 consecutive days in Thy1-H line YFP mouse, plus two-photon imaging for the same region of barrel cortex. (**b**) Central time duration (%) during open-field test. (**c**) Time spent in open arm (%) during elevated plus maze test. (**d**) Imaging of the same dendritic branch on P30 and P45, including eliminated spines (white arrowhead), newly formed spines (yellow arrowhead) and filopodia (asterisk). (**e**) Percentage of spine formation and elimination on P45. Scale bar, 2 μm in **d**. Data were presented as mean±s.e.m. *P<0.05, **P<0.01 and ***P<0.001 using one-way analysis of variance (ANOVA) followed by Tukey *post hoc* test. Refer to Supplementary Table 1 for the number of animals and spines in each group. NS, no significant difference; YFP, yellow fluorescent protein.

We first tested whether treadmill exercise could ameliorate stress-related behavior abnormality. The mice were physically restrained, and were trained on the treadmill for 14 consecutive days, followed by *in vivo* two-photon transcranial microscopy imaging and behavioral tasks (Figure 1a). Both open-field and elevated plus maze tasks showed elevated central zone duration in stressed mice with exercise along with higher open arm duration (*P*<0.001 or <0.05, *post hoc* Tukey test, Figure 1b and Figure 1c), Therefore, treadmill exercise alleviates anxiety-like behavior induced by physical constraint in mice.

Two-photon *in vivo* imaging was performed to examine the dendritic spine dynamics of apical tuft of layer V pyramidal neurons in barrel cortex (Figure 1d). The results showed no significant difference of spine formation among all groups (F(3,20)=0.97, *P*>0.05, one-way analysis of variance; Figure 1e). Physical restrain, however, significantly elevated spine elimination rate, which was restored to normal level by treadmill exercise (*P*<0.001, *post hoc* Tukey test; Figure 1e). These data supported that physical exercise rescued spine elimination rate in stressed mice.

### Stress-induced working memory impairment was ameliorated by physical exercise

**Figure 2:**
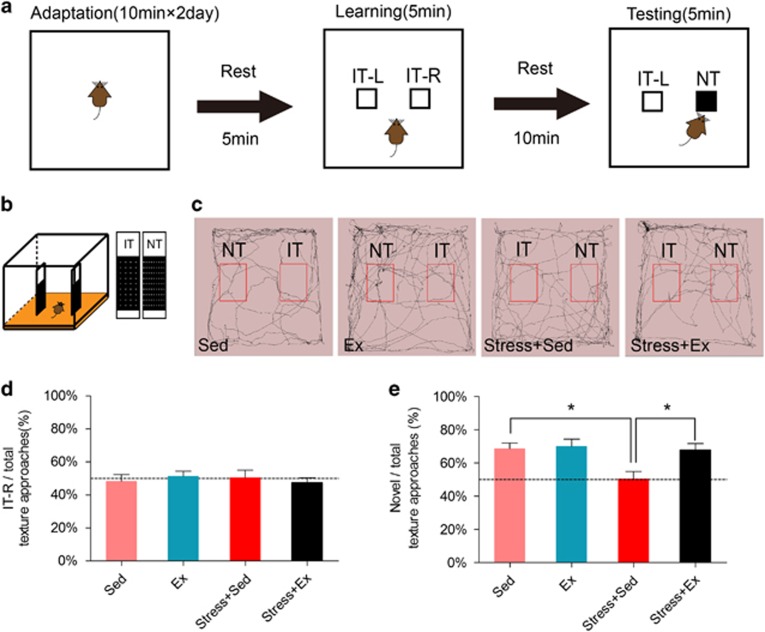
Physical exercise prevented stress-induced working memory impairment in whisker-dependent novel texture discrimination task. (**a**) Schematic illustration of novel texture discrimination task. After a brief adaptation in the test arena (10 min, 2 days) and a short rest (5 min), the mice learned two identical textures (IT-L and IT-R) for 5 min. With different resting intervals (10, 30, 60 and 90 min), the animal was re-placed into the same arena, in which one novel texture (NT) was introduced instead of IT. (**b**) Test apparatus. IT and NT had identical size and color but different textures. (**c**) Movement paths of mice in test phase. Red lines represented arbitrary defined regions in which mice were recorded as approaching the texture. (**d**) Location preference of mice during the learning session. (**e**) Preference of mice for the novel texture in the testing session as calculated by the ratio of novel texture approaching time against total approaching time. Data were shown as mean±s.e.m. *P<0.05 using one-way analysis of variance (ANOVA) followed by Tukey *post hoc* test. *N*=6–7 animals per group.

Stress has been shown to inhibit spatial working memory in prefrontal cortex.^31^ We therefore investigated whether above-mentioned spine plasticity modulation was correlated with memory functions by performing a whisker-dependent novel texture discrimination task on stressed mice receiving treadmill exercise (Figures 2a and b). Neither stress nor exercise changed location preference during initial learning phase (F(3,25)=0.27, *P*>0.05, one-way analysis of variance; Figure 2d). Using a 10 min resting time selected by a pilot study (Supplementary Figure 1), normal mice spent more time exploring novel texture in learning phase. Such behavioral phenotype, however, diminished in stressed mice, and was re-established after treadmill exercise (*P*<0.05, *post hoc* Tukey test; Figure 2e). These data showed that the impaired whisker-dependent working memory loss in stressed mice can be rescued by physical exercise.

### Spine dynamics of barrel cortex was modulated by physical exercise in naive mice

**Figure 3:**
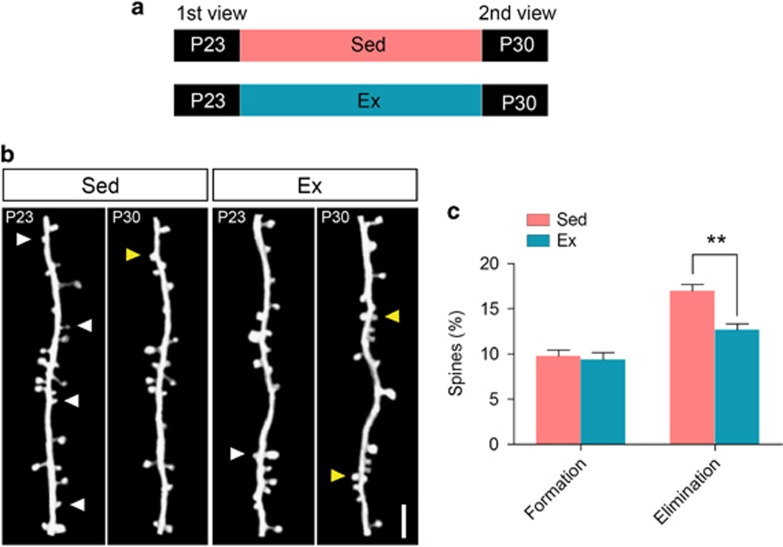
Physical exercise reduced dendritic spines elimination in barrel cortex in naive adolescent mice. (**a**) Schematic diagram showing treadmill exercise (Ex, 1 h per day, 12 m min^−1^) or sedentary housing (Sed) from P23 to P30 and two-photon *in vivo* imaging at the beginning and the end of training. (**b**) Two-photon images showing the same dendritic branch of layer V pyramidal neurons in Barrel cortex, including eliminated spines (white arrowhead), newly formed spines (yellow arrowhead) and filopodia (asterisk). Scale bar, 2 μm. (**c**) Percentage of spine formation and elimination on P30. Data were presented as mean±s.e.m.; NS, no significant difference, **P<0.01 using Student's *t*-test. Refer to Supplementary Table 1 for the number of animals and spines in each group.

Our previous results provided evidences for ameliorating effect of treadmill exercise on stress-induced spine loss and memory impairment. One may ask whether such beneficial effects are just antidepressant response, or global effects independent of mental status. We thus performed another round of 7-day treadmill exercise on P23 adolescent mice plus two-photon imaging (Figure 3a). As agreed with patterns shown in stressed mice (Figure 1e), physical exercise remarkably decreased spine elimination rate (sedentary, 16.97±0.72% exercise, 12.66±0.68%, *P*<0.01, Student's *t*-test; Figure 3c) but not spine formation rate (sedentary, 9.75±0.66% exercise, 9.38±0.78%, *P*>0.05 Student's *t*-test; Figure 3c). Therefore, treadmill exercise can depress spine elimination under both stressed and naive conditions.

**Figure 4:**
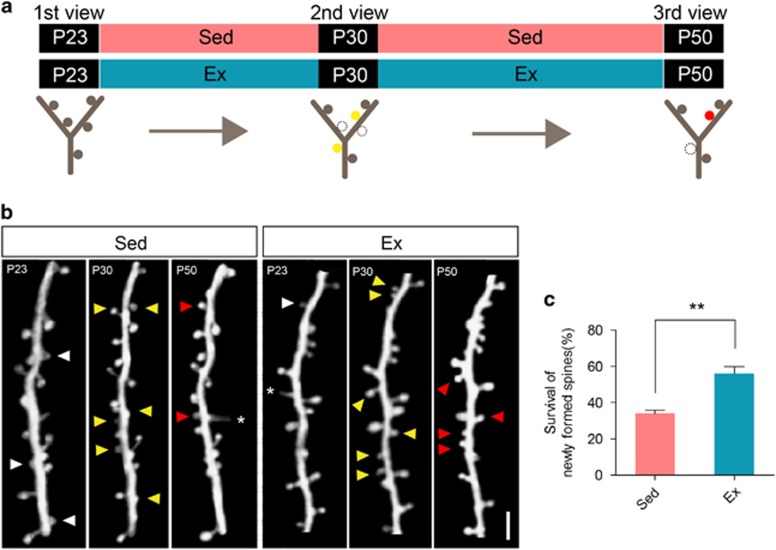
Reduction of dendritic spine elimination was contributed by enhanced newly formed spine survival. (**a**) Following first two-photon *in vivo* imaging of barrel cortex, mice received physical exercise (Ex) or sedentary housing (Sed) from P23 to P50, during which two imaging on the same area were performed. Lower panel: an illustration of one dendritic branch at three time points, showing newly formed spines on P30 (solid yellow circle), which survived (solid red circle) or lost (dash circle) on P50. (**b**) Two-photon imaging of one dendritic branch on P23, P30 and P50. Eliminated spines (white arrowhead), newly formed spines on P30 (yellow arrowhead), survived newly formed spines on P50 (red arrowhead) and filopodia (asterisk) were shown. Scale bar, 2 μm. (**c**) Survival rate of newly formed spines on P50. Data were shown as mean±s.e.m. **P<0.01 using Student's *t*-test. Refer to Supplementary Table 1 for the number of animals and spines in each group.

The formation and elimination of dendritic spine is a dynamic process involving the pruning of newly formed spines in an activity-dependent manner. The suppression of spine elimination thus may be the result of enhanced survival of newly formed spines. We therefore performed a third *in vivo* imaging session after another 20-day treadmill exercise on naive mice (Figure 4a). The observations on P23, P30 and P50 showed subpopulations of dendritic spines that were formed by P30 and were later eliminated or retained by P50 (Figure 4b). Quantitative analysis showed significantly higher survival rate of newly formed spines by P50 in exercise group compared with sedentary group (56.21±3.66% vs 33.96±1.90% *P*<0.01, Student's *t*-test; Figure 4c). These data collectively suggested that treadmill exercise on mice could suppress dendritic elimination via enhancing the survival of newly formed spines.

### Physical exercise prevented stress-induced dendritic spine loss and memory deficits through BDNF–TrkB pathway

**Figure 5:**
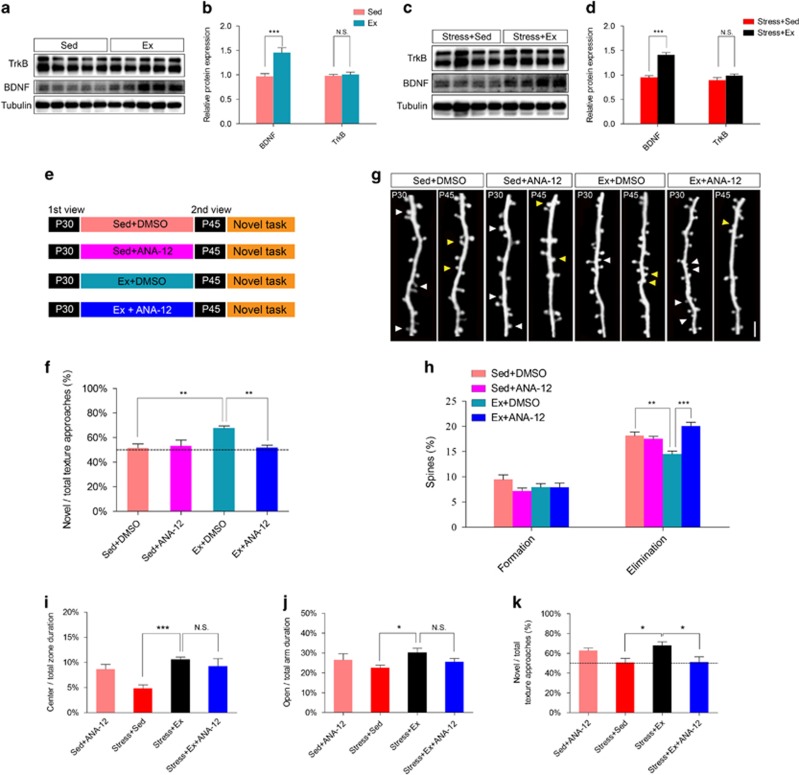
Physical exercise prevented the loss of spines and improved working memory through BDNF–TrkB pathway. (**a** and **b**) Relative protein expression levels of TrkB and BDNF in barrel cortex of naive mouse treated with physical exercise (Ex) or sedentary housing (Sed) from P30 to P45. (**c** and **d**) Relative protein expression levels of TrkB and BDNF in barrel cortex of physically constrained mouse treated with physical exercise (Ex) or sedentary housing (Sed) from P30 to P45. (**e**) Schematic diagram showing two-photon imaging of the same dendritic branch of barrel cortex on P30 and P44, between which mice were sedentary housed (Sed) with DMSO injection or received treadmill exercise (Ex) plus DMSO or ANA-12 injection. (**f**) Preference for the novel texture in discrimination task. (**g**) Two-photon images showing eliminated spines (white arrowhead) and newly formed spines (yellow arrowhead) from P30 to P45. (**h**) Percentage of spine formation and elimination. (**i**) ANA-12 application did not change duration in the central zone of open filed. (**j**) ANA-12 injection did not alter the time spent in open arm during elevated plus maze test. (**k**) ANA-12 injection decreased novel texture recognition ratio of stressed mice even after exercise. Scale bar in **g**, 2 μm. Data were shown as mean±s.e.m.; *P<0.05, **P<0.01, ***P<0.001 using Student's *t*-test in **b** and **d**, or using one-way analysis of variance (ANOVA) followed by *post hoc* Tukey test in **f**–**k**. *N*=6–7 animals per group. Refer to the Supplementary Table 1 for the number of spines in each group. BDNF, brain-derived neurotrophic factor; DMSO, dimethyl sulfoxide; NS, no significant difference; TrkB, tyrosine kinase B.

Physical exercise has been reported to significantly increase the endogenous expression of BDNF,^32^ which is critical for neural development including spine dynamics. BDNF plus its receptor TrkB, were also known to be closely associated with dendritic spine formation.^33^ We found that in both naive and stressed mice, treadmill exercise significantly increased BDNF protein levels in stressed mice (increased by 45.63±9.29% in naive mice and 41.11±4.13% in stressed mice, *P*<0.001, Student's *t*-test; Figures 5a–d), whereas TrkB protein showed similar levels (*P*>0.05). The messenger RNA expression level of BDNF was also elevated in barrel cortex (Supplementary Figure 2). In short, physical exercise stimulated BDNF but not TrkB expression in mouse barrel cortex.

To substantiate the role of BDNF–TrkB pathway in physical exercise-modulated spine plasticity and memory function, we applied ANA-12 to block TrkB pathway. By daily injection of ANA-12 (2.5 mg kg^−1^) for 14 days in conjunction with sedentary or exercise treatment (Figure 5e), improvement of memory retention by physical exercise was totally abolished (*P*<0.001 *post hoc* Tukey test; Figure 5f). Blockade of TrkB pathway also abolished exercise-induced suppression of spine elimination in naive mice (*P*<0.001, *post hoc* Tukey test; Figures 5g and h) but not affected spine formation rate (F(2,11)=1.17, *P*>0.05, one-way analysis of variance; Figures 5g and h). Moreover, we tested ANA-12 effect on stressed mice with exercise. Injection of ANA-12 on exercise-stressed mice did not change their anxiety level, as suggested by unchanged central area duration or open arm duration (*P*>0.05, *post hoc* Tukey test; Figures 5i and j). The blockade of TrkB, as in naive mice, suppressed the exercise-induced novel texture recognition (*P*<0.05, *post hoc* Tukey test; Figure 5k). Such evidences collectively supported that physical exercise suppressed spine elimination in barrel cortex and improved sensory-associated memory in mice in a BDNF–TrkB-dependent manner.

## Discussion

Our previous findings^7, 12^ and other literatures^11, 16, 17^ all supported beneficial effects of physical exercise on hippocampal spinogenesis and neurogenesis, plus rescuing effects of anxiety behavior in stressed animals. Few studies, however, have been performed about the exercise-induced spine plasticity in cortical regions. We thus investigated the effect of treadmill exercise on memory function and spine dynamics of barrel cortex in physically stressed mice. Our results showed impaired novel texture recognition after stress, as consistent with those mice with vibrissae deprivation.^28^ Previous findings have established the necessary role of barrel cortex neurons in encoding whisker afferent inputs.^34, 35, 36^ As stress can induce dendritic spine loss in prefrontal cortex,^2, 37^ it is postulated that stress increases spine elimination in barrel cortex, whereas physical exercise restores spine elimination to normal levels. This hypothesis was later substantiated by two-photon *in vivo* imaging, which clearly showed rescue of stress-induced spine loss in barrel cortex after treadmill exercise. Such improvement of memory function and spine dynamics of barrel cortex by physical exercise exists not only in stressed mice. Using adolescent mice, we found similar behavioral and morphological patterns after 7-day treadmill exercise. Therefore, the improvement of spine turnover and memory function by exercise may be a global effect rather than specific response to stress stimuli.

In revealing the correlation between spine morphology and behavioral phenotypes, previous finding showed that experience-dependent spine plasticity in cortical receptive fields including barrel cortex was accompanied with elevated spine turnover.^38^ Loss of spine plasticity also occurred in somatosensory cortex after long-term sensory deprivation,^20^ whereas fear conditioning increased spine elimination in frontal association cortex.^22^ The auditory cues-paired conditioning, on the other hand, increased spine formation in auditory cortex.^23^ Therefore, different cortical regions may show unique paradigms of spine plasticity even under similar stimulus. In our study, physical stress elevated spine elimination in barrel cortex, whereas treadmill exercise suppressed elimination rate. A correlation analysis showed negative correlation between spine elimination rate and whisker-dependent working memory function (Supplementary Figure 3), as consistent with previous study,^39^ further supporting the necessary role of spine plasticity in the formation and maintenance of whisker-dependent working memory. In a long-term perspective, newly formed spines in barrel cortex are mostly transient under normal circumstance, whereas whisker trimming enhanced spine turnover.^40^ Our results showed retention of newly formed spines by exercise, as similar with those from an object localization task, in which layer 2/3 neurons had potentiated spine growth and stabilization of preexisting spines.^26^ These data further supported the beneficial effects of exercise on cortical related memory function in long-term period.

Morphology is one important factor governing spine plasticity besides its turnover. Dendritic spines can be divided into four subgroups, of which mushroom spines are known to be correlated with memory function in hippocampal neurons.^41, 42^ Subtyping of spines (Supplementary Figure 4) showed that in addition to elevated dendritic spine density compared with sedentary group, exercise increased the percentage of mushroom-like spines from 43.67±0.93% to 55.49±2.00% (*P*<0.05, Student's *t*-test), and decreased thin and filopodia spines (thin: 17.60±0.73% vs 9.15±1.00% fliopodia: 8.47±0.97% vs 3.74±0.52% *P*<0.05 in both the cases, Student's *t*-test). Therefore, treadmill exercise facilitated the maturation of dendritic spines in barrel cortex, in addition to enhancement of the survival of newly formed spines. Aging also dramatically changes spine plasticity and its sensitivity to exogenous stimuli. Dendritic spines undergo rapid growth at initial phase, followed by active turnover and pruning process in adolescent age.^43^ In adult mice (4~6 months old), however, spine turnover rate within a 2-week observation window dropped to 3%~5%.^43^ This study further examined whether physical exercise had any effects on spine dynamics in adult mice. As shown in Supplementary Figure 5, 15-day treadmill exercise did not change either spine formation or elimination rate. These results indicated that our exercise paradigm may only enhance spine turnover in adolescent mice. This is probably due to the vulnerability of spines to exogenous factor during the young adolescent stage, or insufficient working load that was used to induce spine formation or decrease spine elimination in adult mice. In summary, treadmill exercise improved memory via both the retention and maturation of dendritic spines in adolescent mice.

The mechanism underlying exercise-induced spine retention can be illustrated from three aspects. First, stress can modulate BDNF expression and spine formation in different parts of the brain.^44^ This theory received further behavioral evidences as exogenous introduction of BDNF^45^ or its receptor TrkB agonist^46^ prevented stress-induced spatial memory deficits. Second, exercise has been shown to enhance BDNF expression. A series of human studies revealed beneficial effects of physical exercise on BDNF expression.^47, 48, 49, 50^ In major depressive disorder patients, aerobic exercise has been shown to increase serum BDNF level and improve symptoms.^51^ Third, the linkage between exercise and BDNF expressional regulation has also been investigated. Owing to the existence of blood–brain barrier, it is natural to hypothesize that certain peripheral factors may exist and affect central BDNF expression. Ketone body beta-hydroxybutyrate, the metabolite produced after exercise, has been shown to enhance BDNF expression.^52^ Under stressed conditions, hormones including glucocorticoid and corticotropin-releasing hormone can increase spine elimination rate via modulating BDNF levels.^44, 53, 54^ Moreover, as both genetic and epigenetic pathways existed for BDNF expression and related spine morphology regulation,^44, 55, 56^ exercise may modulate epigenetic signals for mediating BDNF levels in the brain. Based on all these information, it is thus highly likely that exercise functioned via certain peripheral factors, which can penetrate blood–brain barrier for activating central expression of BDNF, further suppressing dendritic spine elimination and enhancing survival. Our results supported such hypothesis as treadmill physical stress decreased BDNF protein level in barrel cortex (Supplementary Figure 6), exercise enhanced BDNF protein expression in cortical tissues, whereas the blocking of TrkB pathway reversed exercise-induced suppression of spine elimination and eliminated the memory improvement. Further researches can be conducted to reveal the factor linking exercise and central BDNF expression/spine retention.

In summary, this study demonstrated higher elimination rate of dendritic spines in mouse barrel cortex, accompanied with impaired whisker-dependent memory under stressed condition. Such deficits can be rescued by chronic treadmill exercise via BDNF–TrkB pathway. This study provided evidences for stress-related spine plasticity change, and potential treatment of stress-induced memory deficit using physical exercise, although further studies are required to elucidate underlying neural circuits and molecular mechanisms.
