# Supplementary material for: Treadmill exercise suppressed stress-induced dendritic spine elimination in mouse barrel cortex and improved working memory via BDNF/TrkB pathway
Source: Transl Psychiatry. 2017 Mar 21;7(3):e1069–. doi: 10.1038/tp.2017.41 (PMC5416682; doi:10.1038/tp.2017.41)
Supplement: Supplementary Information [file tp201741x1.docx]

**Supplementary Information**


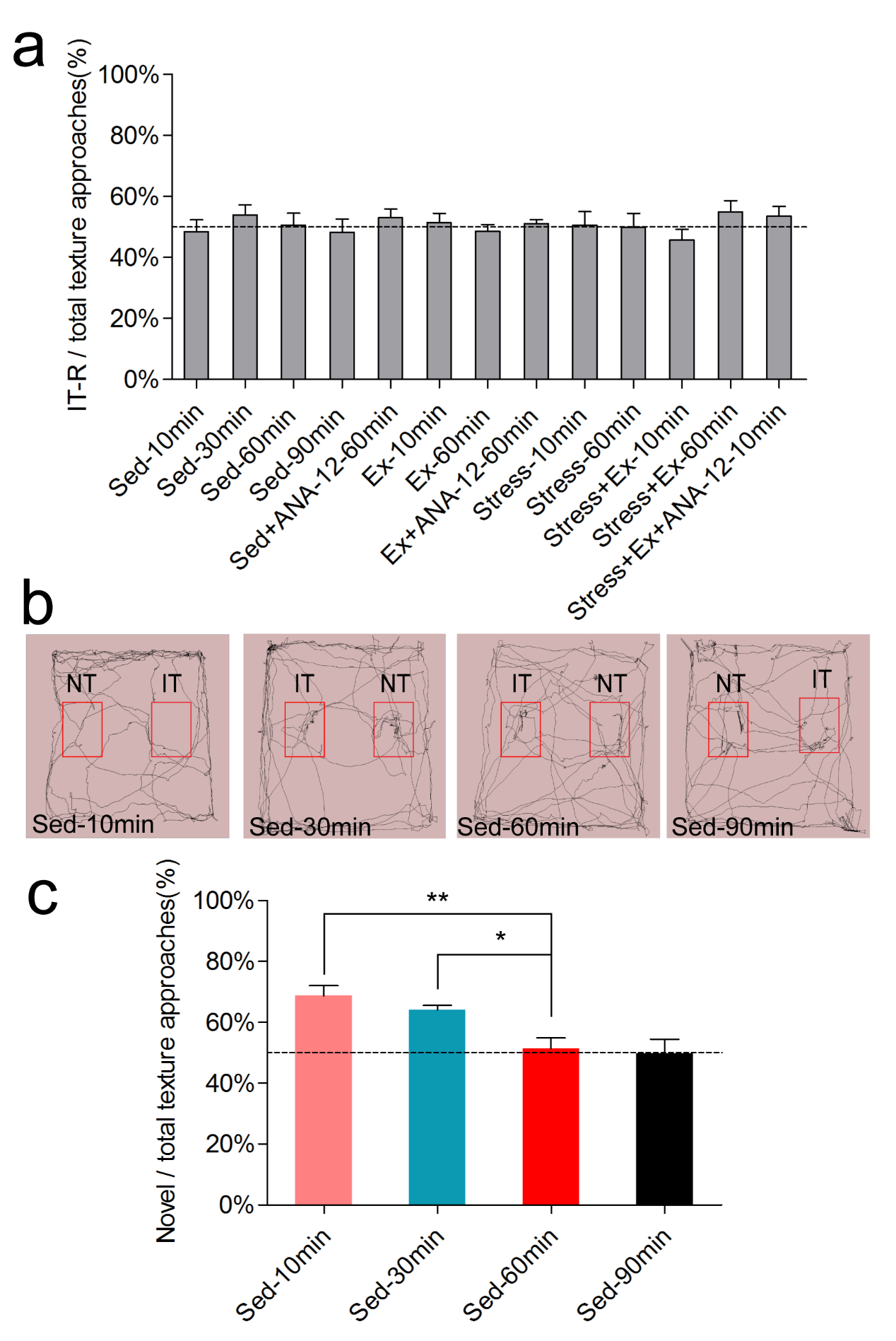


**Supplementary Figure 1 Learning curve during novel texture discrimination task.** (a) No significant difference of place preference was observed across different treatment or various resting time. (b) Movement path of untreated, naïve mice in testing phase after different resting times. (c) Novel texture preferences of sedentary mice after different resting time. *, p<0.05; **, p<0.01 by Tukey post-hoc comparison after one-way ANOVA.


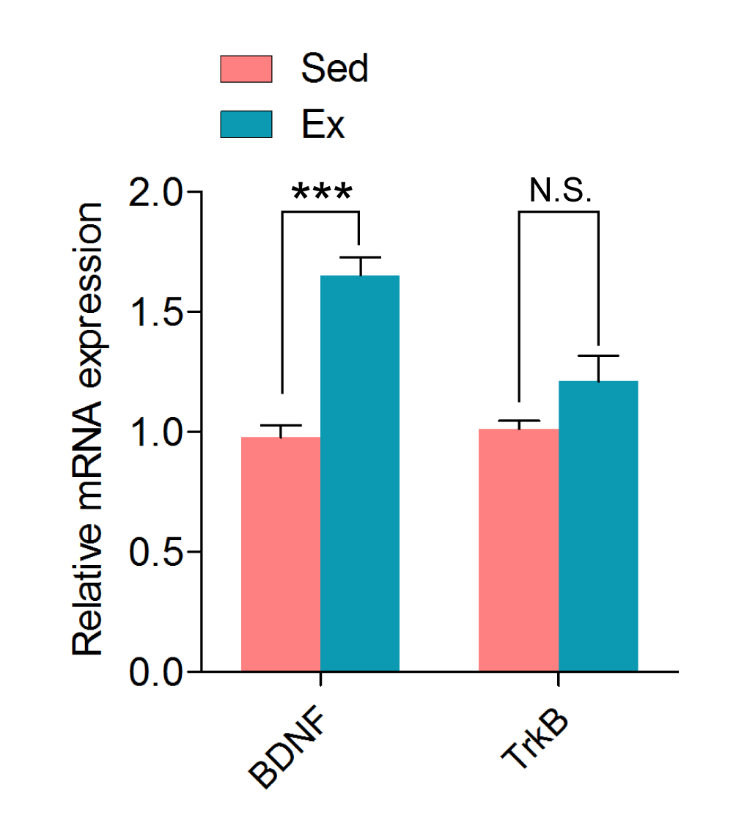


**Supplementary Figure 2 mRNA expression level of BDNF and TrkB in barrel cortex**. N. S., no significant difference. ***, p<0.001 by student t-test.


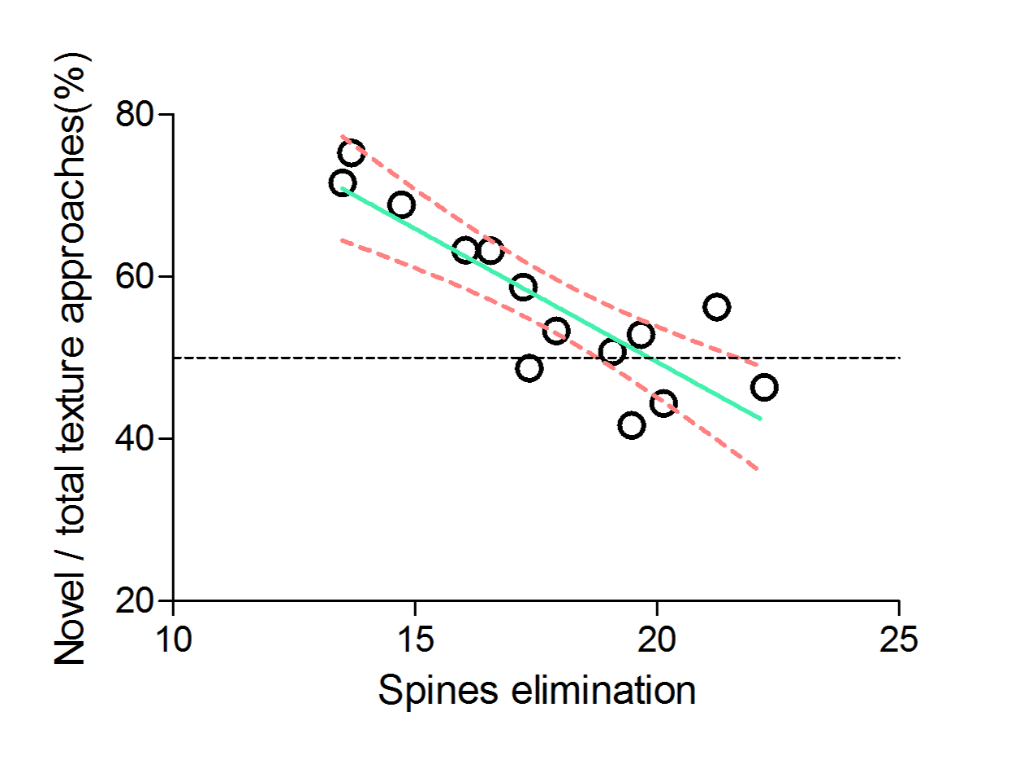


**Supplementary Figure 3 Correlation between spine elimination rate and novel texture preferences.**


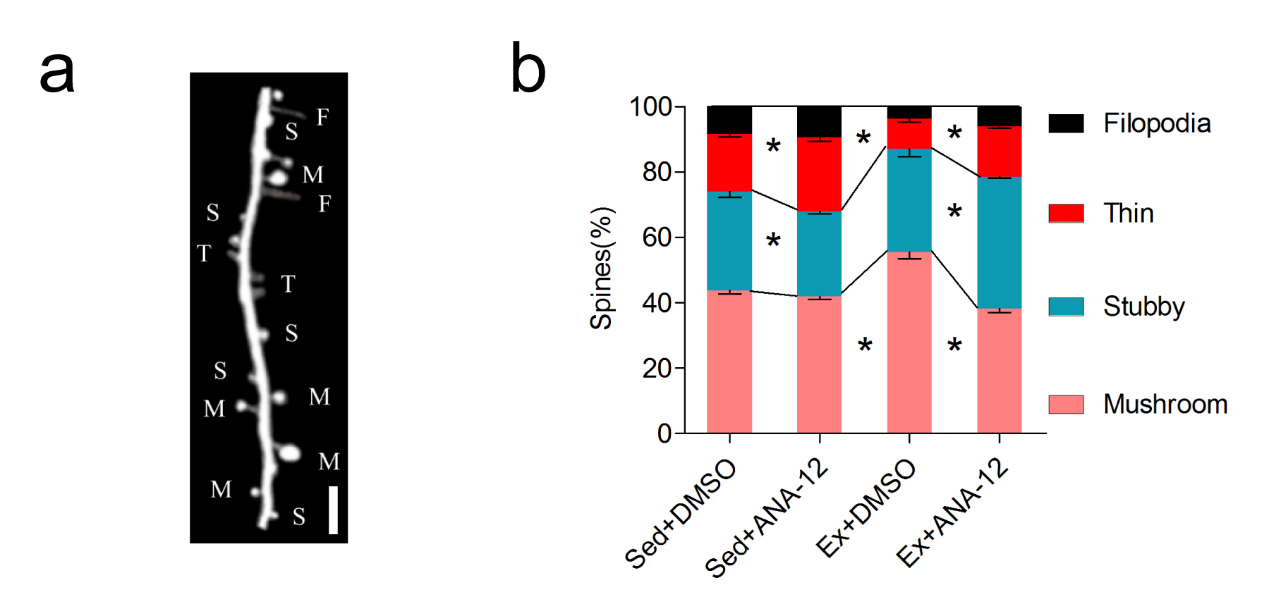


**Supplementary Figure 4 Physical exercises increased matured spine ratio via BDNF/TrkB pathway.** (a) One dendritic branch showing four types of spines: mushroom (M), stubby (S), thin (T) and filopodia (F). (b) Percentage of each type of spines. *, p<0.05 by student t-test.


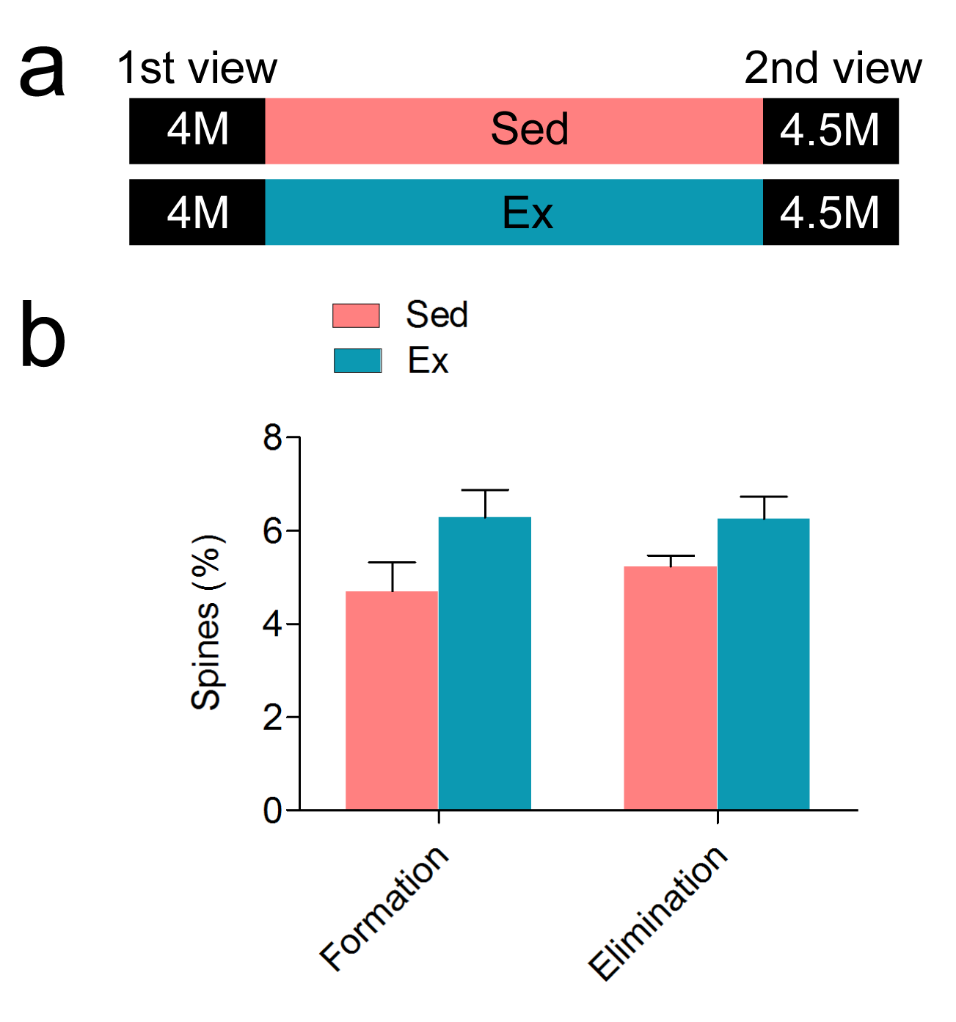


**Supplementary Figure 5 Dendritic spine plasticity in adult (4 month old) mice following physical exercises.** (a) Schematic diagram showing two *in vivo* imaging 4 month and 4.5 month on the same cohort of mice, with intervened sedentary housing (Sed) or treadmill exercises (Ex, 1h per day, 12m/min). (b) Spine formation and elimination rate (%).


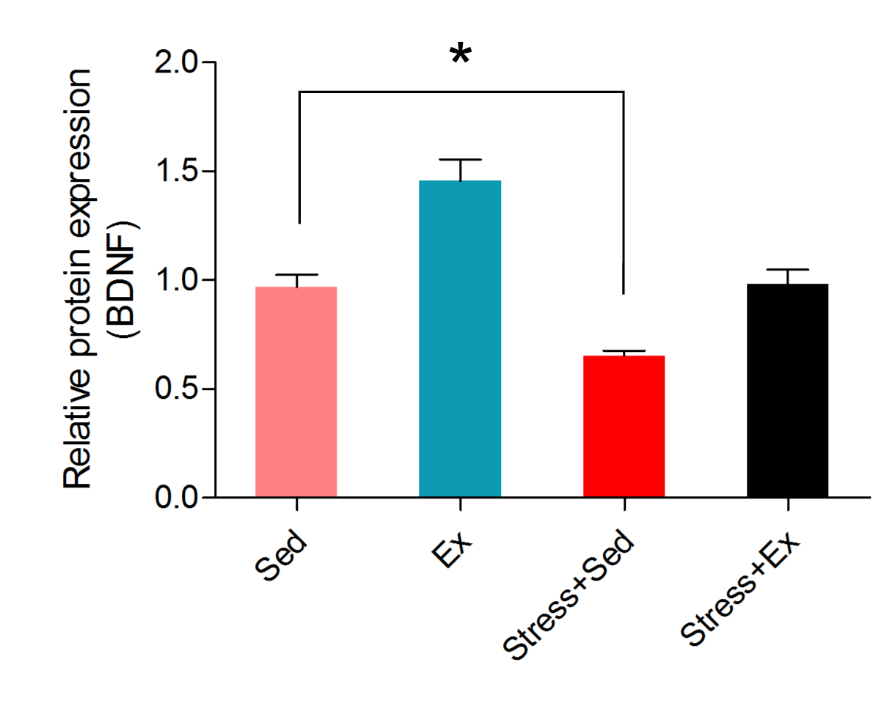


**Supplementary Figure 6 Relative protein expression level of BDNF**. Sedentary (Sed) group was set as the basal level. *, p<0.05 by student t-test.

**Supplementary Table 1 Number of animals, spines and formation/elimination rate in each group.**

| **Spines turnover** | | | | | |
| --- | --- | --- | --- | --- | --- |
| Date interval | Groups | N | 1^st^ spines | Formation%（mean+sem） | Elimination%（mean+sem） |
|  |  |  |  |  |  |
| P23-P30 | Sed | 6 | 1054 | 9.75±0.66 | 16.97±0.72 |
|  | Ex | 4 | 712 | 9.38±0.78 | 12.66±0.68 |
| P30-P44 | Sed | 5 | 744 | 9.47±0.90 | 18.16±0.70 |
|  | Ex | 4 | 656 | 7.94±0.71 | 14.49±0.59 |
|  | Stress+Sed | 6 | 1028 | 7.32±0.83 | 24.59±0.57 |
|  | Stress+Ex | 6 | 1015 | 8.52±1.08 | 16.37±0.55 |
| P30-P44 | Sed+DMSO | 5 | 744 | 9.47±0.90 | 18.16±0.70 |
|  | Ex+DMSO | 4 | 656 | 7.94±0.71 | 14.49±0.59 |
|  | Ex+ANA-12 | 5 | 804 | 7.89±0.85 | 20.03±0.77 |
| >4 month old | Sed | 4 | 619 | 4.70±0.63 | 5.24±0.23 |
|  | Ex | 5 | 800 | 6.29±0.59 | 6.27±0.47 |
| **New spines survival** | |  |  |  |  |
| Date interval | Groups | N | 1^st^ spines | New spines | Survival% |
| P23-P30-P50 | Sed | 4 | 736 | 73 | 33.96±1.90 |
|  | Ex | 4 | 712 | 67 | 56.21±3.66 |
